# Identification of a D-galacturonate reductase efficiently using NADH as a cofactor

**DOI:** 10.1016/j.btre.2022.e00744

**Published:** 2022-06-02

**Authors:** Kaisa E. Peltonen, Peter Richard

**Affiliations:** VTT Technical Research Centre of Finland Ltd., Tietotie 2, 02150 Espoo, Finland

**Keywords:** D-galacturonic acid, EC 1.1.1.365, Cofactor, *Saccharomyces cerevisiae*

## Abstract

D-galacturonate reductases are catalysing the reversible reduction of D-galacturonate to L-galactonate using NAD(P)H as a cofactor. The enzymes are part of two different pathways. One pathway is the fungal pathway for the catabolism of the main compound of pectin, D-galacturonate. The other pathway is a a pathway in plants for L-ascorbic acid synthesis. The previously described naturally occurring enzymes preferably use NADPH as a cofactor. Although certain D-galacturonate reductases, such as the reductases from *Aspergillus niger* or *Euglena gracilis* also accept NADH, their activity is significantly higher with NADPH. We identified in *E. gracilis* a gene, called *gaa1*, coding for a D-galacturonate reductase with similar activities with NADH and NADPH. It is potentially useful for the metabolic engineering of microbes to make use of pectin rich biomass.

## Highlights

• D-galacturonate reductase EC 1.1.1.365 uses NADH efficiently.
• Identification of the D-galacturonate reductase gene in Euglena gracilis.
• A useful tool for the engineering of a pathway for efficient fermentation of D-galacturonic acid.

## 1 Introduction

The use of fossil hydrocarbons for fuels and commodity chemicals harms the environment. This encouraged academic and industrial efforts to identify sources for raw materials that are biobased and sustainable and to develop techniques to convert these raw materials to fuels and chemicals that could replace the petrochemical-derived ones. Underutilized feedstocks that are accessible, available, economic, and do not compete with food resources have gained recent interest. Microbial fermentation using genetically engineered microorganisms is an emerging technique for these conversions. However, the lack of suitable components for genetic engineering, such as genes coding for efficient enzymes hinders the development of efficient fermentation processes.

Pectin-rich residues from the processing of sugar beet, citrus fruit, or apple are an example of biomass that is not used for food and is of low or no value, and therefore a desirable raw material to produce fuels and chemicals. Especially sugar beet pulp and citrus processing waste are of marginal value as animal feed and are abundantly available. Worldwide about 270 million metric tons per year of sugar beet are produced and about 80 million metric tons of citrus. Sugar beet is exclusively used for sugar production and about 40% of the citrus are juiced in centralized factories. The pectin-rich residues are available at the sugar or orange juice factories facilitating the logistics. These pectin rich raw materials have been the target for novel techniques for valorisation. Various approaches were used to engineer microorganisms through metabolic engineering to convert these raw materials to fuels or chemicals [1], but great demand for improved genetic engineering remains. It is important to identify enzymes and their corresponding genes that facilitate the engineering of microorganisms for the use of these underutilized feedstocks. A gene for an efficient NADH using D-galacturonate reductase would be usefull for the engineering because it helps to overcome cofactor imbalances [2].

The D-galacturonate reductase, EC 1.1.1.365, is reducing D-galacturonate to L-galactonate. It uses preferentially NADPH as a cofactor. The enzyme can have different roles. In fungi, it is part of the pathway for D-galacturonate catabolism. D-galacturonate is the main monomer of pectin and the enzyme is active in saprotrophic fungi growing on pectin-rich biomass. In plants, the enzyme is part of one of the several pathways for L-ascorbic acid synthesis. In ripe fruits when pectin is degraded, D-galacturonate is converted to L-ascorbic acid.

### 1.1 Catabolism

There are several pathways for the catabolism of D-galacturonate. In prokaryotes, there are at least two types of pathways, an oxidative, and an isomerase pathway [1]. In the oxidative pathway, D-galacturonate is oxidized in the first step by a NAD requiring dehydrogenase and the pathway provides α-ketoglutarate for the TCA cycle. The isomerase pathway has an isomerase as the first enzyme to produce D-tagaturonate. The pathway produces pyruvate and glyceraldehyde 3-phosphate and in some cases pyruvate and acetate [3]. In fungal microorganisms, D-galacturonate is exclusively catabolized by a reductive pathway. The first step is a reduction to L-galactonate, followed by dehydration and splitting the resulting metabolite to L-glyceraldehyde and pyruvate. The L-glyceraldehyde is reduced by a second reductase to glycerol. Both reductases use NADPH as the preferred cofactor. In fungi, D-galacturonate reductases have been described for several filamentous fungi such as *Trichoderma reesei* [4] and *Aspergillus niger* [5] and in the yeast *Rhodosporidium toruloides* [6]. These enzymes are using exclusively NADPH as a cofactor or as the *A. niger* enzyme GaaA, have a strong preference for NADPH.

### 1.2 Ascorbic acid

In plants, L-ascorbic acid is mainly produced through the Smirnoff-Wheeler pathway but also a set of alternative pathways is in use [7]. One of these alternative pathways starts from pectin and goes through D-galacturonate. D-galacturonate is reduced to L-galactonate by an NADPH requiring enzyme, L-galactonate is cyclized to L-galactono-1,4-lactone which is then oxidized to L-ascorbate. A plant D-galacturonate reductase was first described in *Fragaria x ananassa* to be NADPH specific [8]. The algae *Euglena gracilis* was also described to have a D-galacturonate reductase that was involved in L-ascorbic acid synthesis. This enzyme was different in that it had a preference for NADPH but showed also an activity with NADH. In addition, it showed no activity in the reverse direction with L-galactonate as substrate [9].

Attempts have been made to engineer microorganisms for the utilization of D-galacturonate, the main monomer of pectin. In the yeast, *S. cerevisiae* parts of a bacterial pathway for D-galacturonate catabolism were introduced however a complete and functional pathway has not been reported [10]. The introduction of a fungal pathway in the yeast *S. cerevisiae* resulted in a complete and functional pathway [2,11,12]. A limitation of the fungal pathway is the requirement of NADPH in the first step, the D-galacturonate reductase. An attempt of engineering an NADH-dependent D-galacturonate reductase was successful, however, the efficiency of the mutated enzyme was suboptimal. [13].

## 2 Materials and Methods

### 2.1 Strains

*Escherichia coli* strain TOP10 was used for plasmid construction and BL21(DE3) for bacterial expression. cDNA library screening was done in *E. coli* K-12 JW3063-2, obtained from the Keio Collection [14]. *Saccharomyces cerevisiae* strain CEN.PK2-1D [15] was used for yeast heterologous expression. *Euglena gracilis* strain Z was received as a gift from Marika Tossavainen, University of Helsinki, for the construction of the cDNA library.

### 2.2 Media and culture conditions

*E. coli* was cultured in Luria Broth (LB) medium supplemented with 0.1 g L^−1^ ampicillin, at 37°C, 220 rpm, for cloning and heterologous enzyme expression. Expression cultures were induced with 1 mM IPTG at OD_600_ 0.6-0.8, and incubation was continued at 16 or 30°C for 3 hours or overnight, depending on the expressed enzymes. For yeast transformation, strains were grown in YPD (10 g L^−1^ yeast extract, 20 g L^−1^ peptone, 20 g L^−1^ glucose), at 30°C, 220 rpm. Transformed strains were grown on SCD-URA (uracil deficient synthetic complete medium supplemented with 20 g L^−1^ glucose). *E. gracilis* was grown in Hutner's medium (KH2PO4 0.02 g L-1, potassium citrate • H_2_0 0.04 g L^−1^, MgSO_4_ • 3H2O 0.02 g L^−1^, trypticase peptone 0.6 g L^−1^, yeast extract 0.04 g L^−1^, thiamine 0.04 mg L^−1^, and vitamin B12 0.05 mg L^−1^). Two cultures were used for RNA extraction, one grown at room temperature with natural light, and the other at 30°C covered from light with 1% glucose added to the media. Both cultures were agitated at 200 rpm on an orbital shaker. *E. gracilis* cDNA library was screened in *E. coli* first grown on LB and then D-galacturonate media (1 x M9 salts, MgSO4 2 mM, CaCl2 100 μM, D-galacturonate 10 g L^−1^, L-arabinose 1 g L^−1^, uracil 0.02 g L^−1^, ampicillin 0.1 g L^−1^, chloramphenicol 0.025 g L^−1^, 15 g L^−1^ agar).

### 2.3 Cloning

All plasmids used in this study are listed in Table 1. The ORF for the protein with malate dehydrogenase homology (GenBank GDJR01108896.1) was amplified from the *E. gracilis* cDNA library with primers 5’TGACTCATGATATTCAAGGTTGCCGTCTGTGG and 5’GTACGGATCCTTACTGTTGAACGAACTTGACAC. The primers have 5’ overhangs with the restriction sites for the enzymes *Bsp*HI and *Bam*HI, respectively. The PCR product was digested and ligated to the *E. coli* protein expression vector pBAT4, between the *Nco*I and *Bam*HI restriction sites. The sequence was codon-optimized for *S. cerevisiae* (GenBank OK413949), ordered as a synthetic gene string from Thermo Fisher Scientific (USA), and cloned into the yeast expression vector p2159 between *Eco*RI and *Bam*HI restriction sites in the multiple cloning site.

**Table 1:** List of plasmids used in this study.

Plasmid	Description
pBAT4	*E. coli* expression vector, *Amp ^R^*
pAL17.3	cDNA library plasmid, *Amp^R^*
pTARA	T7 polymerase expressing plasmid, *Cam^R^*
B2159	pXY212, containing TPI1 promoter region, *URA*3 *Amp^R^*
B9681	*E. gracilis* Sequence GDJR01108896.1 as OK413949 in pBAT4
B10444	*E. gracilis* sequence GDJR01031376.1 as OK413948 in p2159
B10445	*E. gracilis* sequence GDJR01031376.1 as OK413948 in pBAT4
B10495	*E. gracilis* sequence GDJR01031376.1 as OK413948 with 6xHIS-tag in pBAT4
B11008	*E. gracilis* sequence GDJR01031376.1 as OK413948 with 6xHIS-tag under TDH3 promoter, *URA*3 *Amp^R^*

The sequence for the *E. gracilis* D-galUA reductase was identified by using a TBLASTN search with *A. niger* reductase gaaA sequence against the *E. gracilis* Transcriptome shotgun assembly (TSA) database. An *S. cerevisiae* codon-optimized sequence was generated (GenBank OK413948) and ordered as a synthetic gene string from Thermo Fisher Scientific. The codon-optimized sequence was cloned by introducing the gene to the multiple cloning site of the pBAT4 plasmid, opened with *Bam*HI and *Nco*I digestion. The C-terminal 6xHIS-tag was introduced by PCR and the product was ligated to a 2µ yeast expression plasmid under a TDH3 promoter. The primers to amplify the ORF and introduce the 6xHIS-tag were 5’AACACACATAAACAAACAAAAGATCTATGGTTGATGTTTTGATGGTTGGTACTGG and 5’CATCAAGATTGCTTTATCTCGAGTTAGGATTTAGTGATGATGATGATGATGAGTTGGTCTAGCTGGAAATGCAACAGC, and the PCR product was ligated to the plasmid backbone using the NEBuilder HiFi DNA Assembly kit. All *E. coli* transformations were performed by electroporation. Yeast plasmids were transformed using the LiAc method.

### 2.4 RNA extraction and cDNA library construction

The *E. gracilis* total RNA was extracted from two cultures grown in light and dark conditions for 6 days. Cells were collected and washed with water, before disrupting a portion of the combined pellet with 0.5 ml 0.5 mm glass beads in 1 ml TRI Reagent using the Precellys 24 tissue homogenizer (Bertin Technologies, France). The disruption of the cells was conducted twice at 5200 rpm, 30 s, cooling the tube on ice between the beatings. Cell debris was removed by centrifugation at 14 000 rpm, 5 min, 4°C, and the supernatant was transferred to a new 2 ml tube. RNA was separated from the lysate by mixing in 200 μl of chloroform and separating the sample solution into three phases by centrifuging at 12 000 g, 15 min, 4°C after a 2-minute incubation at room temperature. The upper aqueous layer, containing the RNA, was transferred carefully to a new tube, and RNA was precipitated by adding 500 μl of isopropanol and mixing by inverting the tube gently a few times. The sample was incubated at room temperature for 10 minutes, after which the RNA was pelleted by centrifugation at 12 000 g, 10 min, 4°C, and the supernatant was removed. The pellet was washed with 1 ml of cold 75% EtOH, vortexing the tube. RNA was re-pelleted by centrifugation at 7500 g, 5 min, 4°C, after which the ethanol was removed. The pellet was dried for 30 minutes at room temperature, and the extracted RNA was solubilized in 50 μl DMPC treated water, and purity and RNA concentration was assessed with NanoDrop 2000 (Thermo Fisher Scientific). The RNA was sent for cDNA library construction to Evrogen, Russia. The cDNA library was constructed in a pAL17.3 plasmid and provided cloned into *E. coli* (XL1-blue). A 10-μl inoculum of the library stock was grown for 4 hours in LB supplemented with ampicillin 100 μg mL^−1^ before the extraction of the plasmid library. The purified library was used as the template for *in vivo* screening.

### 2.5 In vivo screening of the *E. gracilis* cDNA library

The expression of the cDNA library was achieved by co-transforming an AraC-PBAD-regulated T7 RNA polymerase coding plasmid pTARA [16] with the T7 regulated cDNA plasmid in the expression strain. The screening was done in an *E. coli* K-12 strain with deletions of the D-galacturonate isomerase gene *uxaC* and the arabinose pathway. The deletion of the *uxaC* prevents the natural catabolism of D-galacturonate in *E. coli*, hence the expression of the reductase from the cDNA library would restore the ability to utilize D-galacturonate. The deletion of the arabinose pathway allowed the induction of the pTARA plasmid with a small amount of arabinose in the growth media.

The strain transformed with the pTARA plasmid was transformed again with the cDNA library and grown overnight on five plates of LB agar supplemented with ampicillin and chloramphenicol. The transformants were then collected from the plate by suspending the colonies into 1 ml of sterile DDIW. The cell suspensions were pooled together, washed, and diluted 1:1000 before plating on the D-galacturonic acid agar medium with added L-arabinose and antibiotics. The appearance of colonies was checked after incubation of 7-14 days at room temperature. The appeared colonies were streaked on LB agar with ampicillin, but without chloramphenicol to lose the pTARA plasmid. The cDNA plasmid for each colony was then extracted and sequenced. Primers annealing to the pAL17.3 plasmid upstream (5’AATACGACTCACTATAGGG) and downstream (5’ATTTAGGTGACACTATAGAAATCTC) of the insert amplified the cDNA sequence. The sequences obtained were compared to the N-terminal sequence of the enzyme described by Ishikawa et al. and to the *E. gracilis* Transcriptome Shotgun Assembly database. The identified partial match (GenBank GDJR01108896.1) was expressed in *E. coli* and *S. cerevisiae* and assayed for D-galacturonate reductase activity.

### 2.6 Preparation of cell lysates

Bacterial and yeast cell lysates were prepared by different methods. Cells from induced *E. coli* cultures were collected by centrifugation at 5000 rpm, 5 min at 4°C, and washed with water. The pellet was suspended in 25 ml of buffer containing 50 mM Tris-HCl pH 7.5, and protease inhibitor Complete EDTA free (Roche, Switzerland). Cells were disrupted by sonicating 8 × 20 s on ice, with 50 s intervals in between each sonication. Cell debris was removed by centrifugation at 14 000 rpm for 15 minutes at 4°C.

Cell lysates of *S. cerevisiae* cultures were prepared by mechanical disruption of cells. The cells of 50 ml overnight cultures were collected by centrifugation and washed with water. The washed pellet was suspended in 1 ml 0.1 M Na-phosphate buffer pH 7, and 1 ml of the suspension was transferred to a tube with 0.5 ml glass beads (Ø 0.5 mm) and protease inhibitor. Cells were disrupted with Precellys homogenizer by beating 3 × 30 s at 6500 rpm, cooling the tubes on ice for 5 minutes between each round. The lysate was cleared by centrifugation, and the cleared lysate was used for enzyme assays.

### 2.7 Purification of His-tagged enzyme

Cleared cell lysate from ten 50 ml overnight yeast cultures were passed through a 0.2 µm sterile syringe filter and diluted with twice the original volume of binding buffer containing 20 mM sodium phosphate, 0.5 M NaCl, and 40 mM imidazole, pH 7.4. The column HisTrap HP 1 ml (GE Healthcare, USA) was equilibrated with the binding buffer, on the Äkta start protein purification system (GE Healthcare). The enzyme was eluted with a gradient from 0 to 100% with 10 ml of elution buffer (20 mM sodium phosphate, 0.5 M NaCl, 500 mM imidazole, pH 7.4), and the elute was collected in 0.5 ml fractions. The purity and size of the enzyme were estimated on SDS-PAGE using 4-20% Criterion TGX precast gel (Bio-Rad, USA). Total protein concentration in the purified fraction was assayed using Bio-Rad Protein Assay with bovine γ-globulin as reference protein.

### 2.8 Enzyme assays

The enzyme activities of the putative D-galacturonate reductases were assayed in the forward direction by following the decrease in absorbance at 340 nm caused by the oxidation of NAD(P)H. The L-galactonate activity was assayed in the reverse direction with NAD(P). Malate dehydrogenase activity of the protein coded by sequence with ID GDJR01108896.1 was assayed in the reverse direction using oxaloacetate and NADH as substrates. The assays with crude cell extract were performed with Ultrospec 2100 pro spectrophotometer (GE Healthcare) in a 1 ml cuvette at room temperature. The substrate concentration was 2.5 mM for D-galacturonate, 1 mM for L-galactonate, and 5 mM for oxaloacetic acid while the cofactor concentration was 0.2 mM. The reaction was followed for 5 minutes and absorbance was measured at 30-second intervals.

Enzyme kinetics of the *E. gracilis* reductase were assayed using the Konelab Arena 20XT system (Thermo Fisher Scientific). The affinity for different substrates and cofactors was tested by varying the concentration of each substrate in the reaction mix. For D-galacturonate affinity and for the L-galactonate affinity, the concentration ranged from 0.1 mM to 5 mM, with 1 mM cofactor concentration. The cofactor affinity was assayed with concentrations ranging from 0.01 to 1 mM, with 100 mM D-galacturonate. The concentration of the purified enzyme in the reaction mix was 12.5 µg mL^−1^. The reaction mix was buffered with 0.1 M Na-phosphate buffer pH 7, and the reactions were performed at 30°C.

## 3 Results

An enzyme with D-galacturonate reductase activity that uses NADH as a cofactor was previously described in *E. gracilis*. The enzyme was purified, and an N-terminal amino acid sequence was determined. This N-terminal sequence was FKVAV?GAAAGIGQPL [9]. When searching the *E. gracilis* Transcriptome Shotgun Assembly (TSA) at the NCBI database (www.ncbi.nlm.nih.gov) we could not find an exact match.

In the next approach to identify the D-galacturonate reductase from *E. gracilis* we screened a cDNA library in *E. coli*. cDNA was made from *E. gracilis* grown in light or the dark on glucose. The *E. coli* strain used for screening had a deletion in the *uxaC. E. coli* uses the isomerase pathway for D-galacturonate catabolism and UxaC is catalyzing the reaction from D-galacturonate to D-tagaturonate. Strains with an *uxaC* deletion cannot grow on D-galacturonate. The strain is suitable to screen for a D-galacturonate reductase catalyzing the reaction from D-galacturonate to L-galactonate since *E. coli* can grow on L-galactonate [17]. Transformants were first grown on LB medium and then transferred to the D-galacturonate medium. The functionality of the assay was first tested with a cDNA library from *Aspergillus niger* grown on D-galacturonic acid. With this screen, we expected to find the *A. niger* D-galacturonate reductase gene gaaA. We screened 10^6^ clones of the *A. niger* cDNA library for growth on D-galacturonate and identified the reductase gene gaaA in 10 transformants exhibiting growth after one week of incubation at room temperature, showing that the screen was functional. Screening the *E. gracilis* cDNA library we identified about 100 transformants growing very slowly after 1-2 weeks. Finally, we found a sequence matching the described N-terminal sequence. The transcribed RNA sequence with the GenBank identifier GDJR01108896.1 coded for a hypothetical protein with the N-terminal sequence MPFKVAVCGAAGGIGQPL, which had a mismatch only in one amino acid. The protein had the highest similarities with malate dehydrogenases. The sequence corresponding to the 318 aa ORF starting with the above N-terminal sequence was amplified from an *E. gracilis* cDNA library and cloned and expressed in *E. coli*. For yeast expression, a codon-optimized synthetic DNA string was used for cloning. The expression of this enzyme in *E. coli* or *S. cerevisiae* did not result in detectable D-galacturonate reductase activity in the cell lysates. However, the enzyme did have malate dehydrogenase activity assayed from *E. coli* crude extract with oxaloacetate and NADH.

In an alternative approach to identifying in *E. gracilis* an NADH D-galacturonate reductase we searched the genome for homologs of GaaA, the D-galacturonate reductase from mold the *A. nige*r. Using the NCBI database we made a tblastn search (protein to translated nucleotide) using the amino acid sequence of the *A. niger* GaaA as a query sequence to find homolog sequences in the *E. gracilis* TSA database. The closest homolog had the GenBank identifier: GDJR01031376.1, the open reading frame was between the nucleotides 1371-109 and coded for a protein with 420 amino acids. The N-terminal sequence had no similarities to the sequence from the purified *E. gracilis* protein. Attempts to clone the gene from the *E. gracilis* cDNA library were not successful. The gene was then custom synthesized and expressed in both *E. coli* and *S. cerevisiae*. We were able to detect D-galacturonate reductase activity in the crude cell extracts of both *S. cerevisiae* and *E. coli*. The enzyme catalyzed also the reverse reaction with L-galactonate as a substrate.

**Fig. 1:**
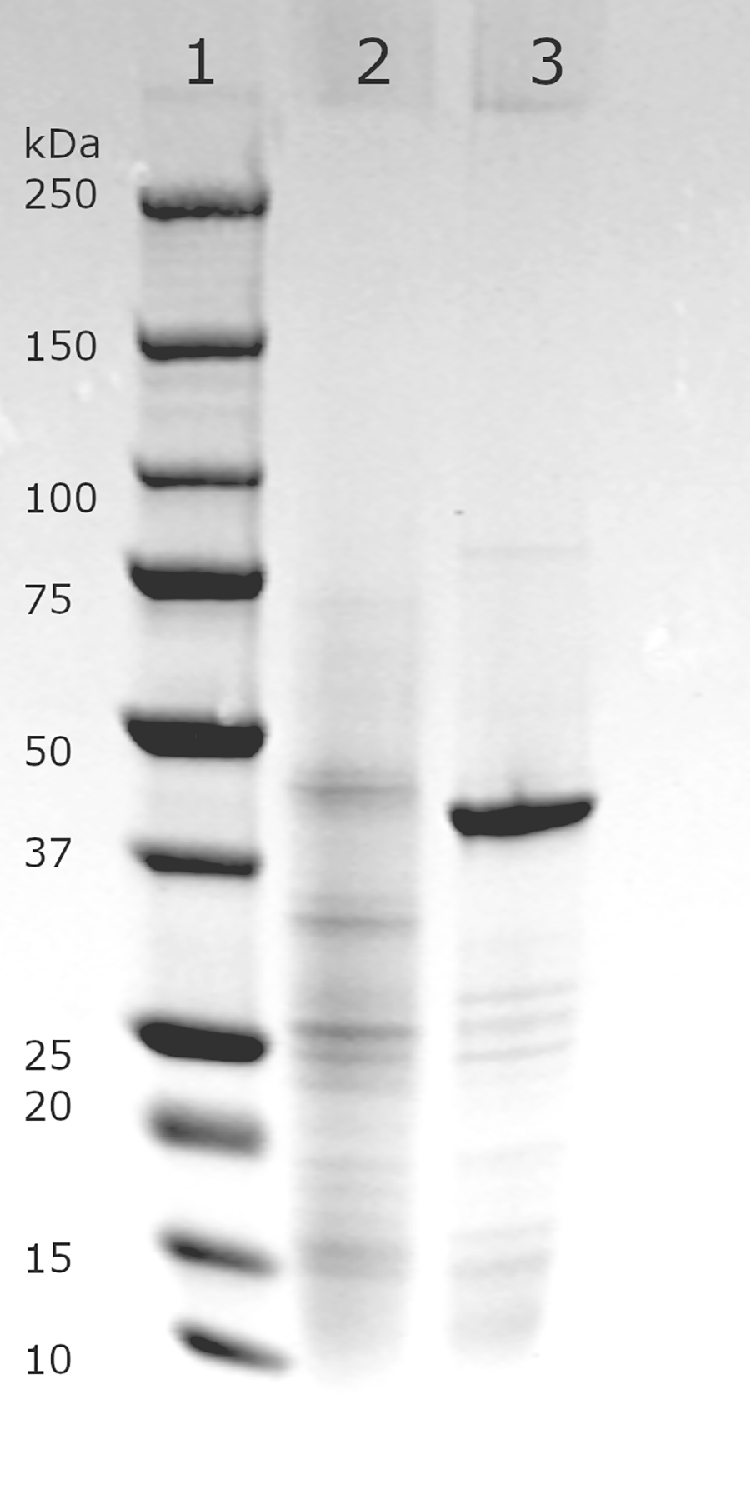
SDS-PAGE gel of purified Gaa1. Lane 1 is marker Precision Plus Protein Standard (Bio-Rad), lane 2 purified Gaa1 enzyme.

**Fig. 2:**
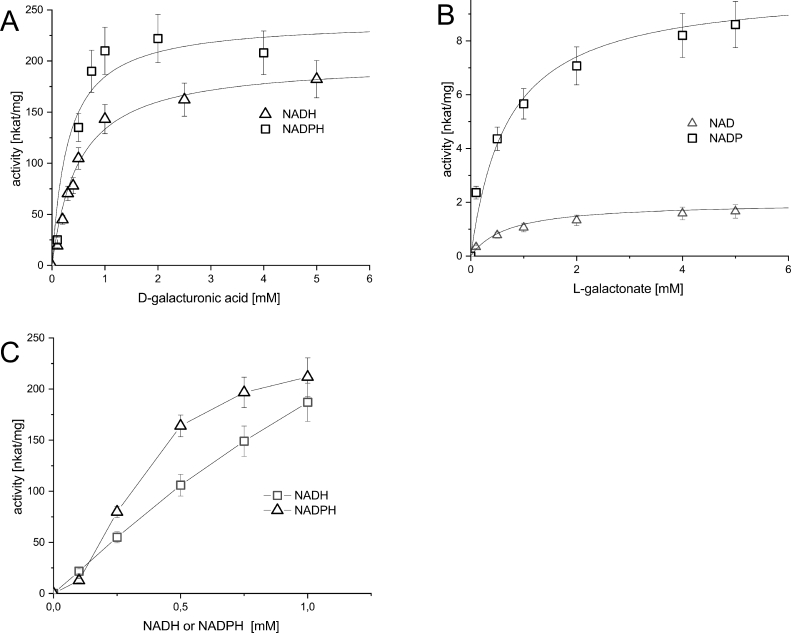
Kinetics of the purified *E. gracilis* Gaa1 reductase. Depicted enzyme activities for A) D-galacturonate with constant NADPH or NADH of 1 mM B) L-galactonate with constant NADP or NAD of 1 mM C) NADPH or NADH with constant D-galacturonate of 100 mM

To characterize the enzyme we added a C-terminal 6xHIS tag. The expression was tried in *E. coli* using the pBAT4 vector containing the T7 promoter [18] and the *E. coli* strain BL21 (DH3). We tried several conditions with different IPTG induction times (3 h to overnight) and temperatures (16, 30, and 37°C), but could not detect D-galacturonate reductase activity in the crude cell extract. We then expressed the His-tagged protein in *S. cerevisiae* using a multicopy yeast expression plasmid. After detecting activity in the crude cell extract, we continued to purify the protein by IMAC using a Ni Sepharose column. The size of the protein was calculated to be ∼45 kDa, and the purified protein showed a similar size band on SDS-PAGE (Fig. 1). This size is different from the D-galacturonate reductase purified from *E. gracilis* (38-39 kDa) [9] but similar to the *A. niger* reductase (44 kDa) [5]. The purified enzyme was used to measure enzyme kinetics, and the resulting Michaelis-Menten curves are depicted in Fig. 2. In Fig. 2 A, the initial reaction rates are shown at different D-galacturonate concentrations when NADH or NADPH was used as the cofactor. The resulting Michaelis-Menten values are in Table 2. Fig. 2 B shows the reverse reactions at various L-galactonate concentrations using NAD or NADP as the cofactor. Fig. 2 C shows the initial rates of the forward reaction at varying NADH or NADPH concentrations. For NADH and NADPH Michaelis-Menten constants could not be estimated since substrate saturation was not reached.

**Table 2:** Activities and affinities of characterized D-galacturonic acid reductases to substrates D-galacturonic acid (GalUA), L-galactonic acid (L-galA), and cofactors.

Substrate	nkat/mg	Kcat	Km (mM)	Kcat/Km	organism
GalUA (NADPH)	240	10.8	0.3	36 000	*E. gracilis* (this study)
GalUA (NADH)	200	9.0	0.5	18 000	
NADPH	-	-	-	-	
NADH	-	-	-	-	
L-galA (NADP)	10	0.45	0.7	650	
L-galA (NAD)	2	0.09	0.7	130	
					
GalUA (NADPH)	145	6.88	0.175	39 289	*A. niger*[5]
GalUA (NADH)	115	5.45	7.11	767	
NADPH	150	7.11	0.036	197 222	
NADH	105	4.98	0.326	15 273	
L-galA (NADP)	3.5	0.16	-	-	
L-galA (NAD)	1.7	0.08	-	-	
					
GalUA (NADPH)	835	39.6	4.4	9 000	*T. reesei*[4]
GalUA (NADH)	-	-	-	-	
NADPH	668	31.7	0.045	704 000	
NADH	-	-	-	-	
L-galA (NADP)	33.4	1.58	4	395	
L-galA (NAD)	-	-	-	-	
					
GalUA (NADPH)	553	26.2	7	3 750	*R. toruloides*[6]
GalUA (NADH)	-	-	-	-	
NADPH	-	-	-	-	
NADH	-	-	-	-	
L-galA (NADP)	2940	139	5.8	23 965	
L-galA (NAD)	-	-	-	-	

**Fig. 3:**
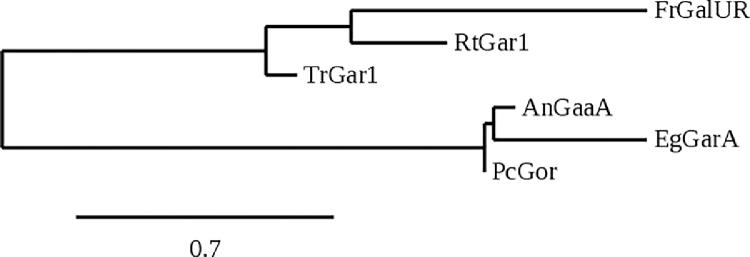
Phylogenetic analysis of different D-galacturonate reductases. AngaaA is GaaA from *Aspergillus niger*, Pcgor is Gor from *Penicillium camemberti*, the Bcgar2 and Bcgar1 are from *Botrytis cinerea*, Eggaa1 is Gaa1 from *Euglena gracilis*, Ndgar1 is Gar1 from *Naganishia diffluens*, Trgar1 is Gar1 from *Trichoderma reesei*, Rtgar1 is Gar1 from *Rodosporidium toruloides*, and Fxagalur and Rrgalur are Galur from *Fragaria x ananassa* and *Rosa roxburghii*, respectively.

For the phylogenetic analysis of different D-galacturonate reductases, the Maximum Likelihood method and the Whelan and Goldman model were used [19] based on the amino acid sequences of the reductases. The tree (Fig. 3) was generated with 1000 bootstraps and is drawn to scale, with branch lengths measured in the number of substitutions per site. The percentage in which the taxa is clustered is shown next to the branches. The protein sequences were aligned using ClustalW with a gap opening penalty of 3 and a gap extension penalty of 1.8, and the evolutionary analysis was conducted in MegaX [20]. AngaaA is GaaA from *Aspergillus niger,* Pcgor is Gor from *Penicillium camemberti*, the Bcgar2 and Bcgar1 are from *Botrytis cinerea*, Eggaa1 is Gaa1 from *Euglena gracilis*, Ndgar1 is Gar1 from *Naganishia diffluens,* Trgar1 is Gar1 from *Trichoderma reesei*, Rtgar1 is Gar1 from *Rhodosporidium toruloides*, and Fxagalur and Rrgalur are Galur from *Fragaria x ananassa* and *Rosa roxburghii*, respectively.

## 4 Discussion

The D-galacturonate reductase, EC 1.1.1.365, is present in eukaryotes, fungi and green plants, and algae. In fungi, it is in the eukaryotic degradation pathway of D-galacturonate, a pathway that is distinctly different from all prokaryotic D-galacturonate degradation pathways. D-galacturonate reductase has been identified in the filamentous fungi *Trichoderma reesei* [4], *Aspergillus niger* [5], *Botrytis cinerea* [21] *Penicillium camemberti* [22] and in the yeasts *Naganishia diffluens* [23] and *Rodosporidium toruloides* [6]. The enzymes from these fungi can only use NADPH as a cofactor. An exception is the GaaA from *A. niger*. This enzyme can also use NADH, however, the affinity for D-galacturonate is lowered when NADH is the cofactor. The Km for D-galacturonate with NADH is 7.11 mM compared to a Km for D-galacturonate with NADPH of 0.175 mM. Since also the Km for NADPH (0.036 mM) is lower than the Km for NADH (0.326 mM) [4] the *A. niger* GaaA is using mainly NADPH as a cofactor (Table 2).

Green plants have also a D-galacturonate reductase. It was described in *Rosa roxburghii* [24] and *Fragaria x ananassa* [8]. Here the enzyme is involved in the metabolism of D-galacturonate that is derived from pectin, for example in ripening fruits. D-galacturonate is here not catabolized but converted to L-ascorbic acid. The enzyme is part of one of the several pathways for L-ascorbic acid synthesis in green plants. Only the *Fragaria x ananassa* enzyme was characterized and it was specific for NADPH as a cofactor. In algae, the D-galacturonate reductase is also part of the pathway for the synthesis of L-ascorbic acid from D-galacturonate. The enzyme characterized in this work is most similar to the D-galacturonate reductases from mold and less similar to the L-ascorbate related plant enzymes from *F. x ananassa* and *R. roxburghii* (Fig. 3). The phylogenetic analysis of the reductases is in line with previous publications categorizing the reductases in two different families, the NAD(P)-binding Rossman fold family of reductases [5,22,23] and the aldo-keto reductases [4,8].

The enzyme that was previously purified from *E. gracilis* and characterized had also activity with NADH. The relative activity with NADH was 17.7% compared to the activity with NADPH [9]. The enzyme described in this communication is different since it always has a similar activity with NADH compared to the activity with NADPH (Table 2). The enzyme characterized in this communication is from the same organism *E. gracilis* strain Z as previously described by Ishikawa et al. [9], however it has several features that are different. The N-terminal amino acid sequences are different and some of the kinetic properties differ. Another difference was that the enzyme purified by Ishikawa et al. was not katalysing the reverse reaction with L-galactonic acid and NADP as substrates. This suggests that the organism might have more than one gene coding for a D-galacturonate reductase.

Several activities for the engineering of microorganisms to use D-galacturonate for the production of fuels or chemicals have been reported. The gene in this communication coding for an enzyme that efficiently uses NADH for D-galacturonate reduction. This is a usefull element for the engineering of microorganisms for the efficient conversion of pectin rich biomass to usefull chemicals.

## Funding

This research was funded by the Academy of Finland, grant number 311743 (ERASynBio 2016: YEASTPEC).

## Ethics approval and consent to participate

Not applicable

## Consent for publication

Not applicable

## Availability of data and materials

All the data used is publicly available as specified in the manuscript.

## Authors' contributions

The authors planned, designed, performed, and designed the experiments and wrote the manuscript.

## Declaration of Competing Interest

The authors declare that they have no known competing financial interests or personal relationships that could have appeared to influence the work reported in this paper.
